# “I’ll Choose My Own Way”: Delinquent Girls and Boys in Search of Gender Hegemony

**DOI:** 10.1007/s10612-022-09607-2

**Published:** 2022-02-10

**Authors:** Armelle Weil

**Affiliations:** 1 HETS, Rue Prévost-Martin 28, CP. 80, 1211 Geneva 4, Switzerland; 2 University of Lausanne, Quartier Unil-Mouline, 1015 Lausanne, Switzerland

## Abstract

This article analyzes juvenile delinquency through the concept of “gender projects.” It argues that delinquency makes the embodiment of specific masculinities and femininities possible, and thereby contributes to “gender achievement” (or “gender accomplishment”). Drawing on in-depth interviews conducted with Swiss juvenile offenders and using Raewyn Connell’s (1987, 1995) notion of “hegemonic masculinity,” this article examines “gender projects” that boys and girls pursue through a variety of offenses and trajectories in the criminal justice system. By inscribing the youth’s delinquent trajectories in social space and by paying attention to the intersection of power relations they face, this article discusses how delinquency can be a tactic to sustain, produce or overcome gender hegemony.

## Introduction

A variety of methods, theoretical approaches and foci of inquiry have been adopted to explain the greater percentage of young men’s offenses and the lower, albeit increasing, presence of young women in crime. Indeed, scholars from around the world tend to agree that the theoretical and empirical analyses of juvenile delinquency support a “gender gap”—one in which boys offend at higher rates and with greater frequency than girls (Lanctôt and Le Blanc 2002). Studies show that female and male delinquency is differentiated along gender lines both quantitatively and qualitatively—for example, with the type of offenses and contexts in which they are committed, or in the domestic environments of young people.^1^ An extensive literature search identifies multiple “discriminatory” factors and stages, including gender-biased police identification, extra-judicial management of girls’ deviance and, more generally, a gendered socialization that affects pathways to crime (Chapple and McQuillan 2019; Chesney-Lind and Pasko 2013). Studies on “gender and judging” have established that sentencing is subject to a gender bias, in adults and minors alike (Bontrager et al. 2013; Schultz and Shaw 2013). Regarding young offenders, several authors point to the existence of a dualistic model that hands out punishment to boys but treatment to girls; boys are perceived as violent and a danger *to others*, whereas girls are perceived as “troubled” and a danger *to themselves* (Henriksen 2018; Moore and Padavic 2010; Niget 2012). Moreover, boys and girls seem to be characterized by two distinct stereotypes of delinquents: the masculine attacker and the unruly girl, respectively (Niget 2012).

On the one hand, most studies base their findings on analyses of how individual youths are treated by the courts or criminal institutions, thus leaving unexplored the question of the differences between young men and women before labeling or sentencing by the justice system. On the other hand, studies on juvenile delinquent trajectories are predominantly quantitative, thus providing little insight into the lived experiences of youths (Carlsson 2013). By way of response, this article will shift focus by analyzing the gender-based differentiation articulated by the youths themselves, adopting a critical gender perspective. I argue that part of the explanation of the gender gap lies in the youth’s “gender projects” (Connell 1987, 1995), defined as “practically accomplished constructions of gender that emerge over time through interactions with others, self-reflection, and responses to socially historic features of contemporary life” (Lyng and Matthews 2007: 84). By focusing on the youth’s “gender projects,” I will examine how delinquency contributes to “gender achievement.” Using in-depth interviews with young people who have committed offenses, I posit—as James Messerschmidt (2004) did for violence—that certain masculinities and femininities are enacted through delinquent acts. These offenses allow young people to realize expectations linked to their “gender projects,” which are also expressed and reinforced by parents, peers, and criminal justice actors. In other words, delinquency, in addition to being dependent on sentencing and labeling, as mentioned above, can be analyzed as a tool for negotiating and performing gender.

This article hopes to contribute to the research on juvenile delinquency and gender. Its aim is to put forward the voices of youth who experience delinquency, to question normative definitions of crime, and to challenge biological or deterministic explanations of the gender gap in offending. I begin by presenting the theoretical framework and the methodology used, based on my ongoing doctoral research. I then present the trajectory of two youths (a boy and a girl) whom I interviewed, and I discuss the interactions between their delinquent practices and discourses, as well as the gender identity to which each aspires. I then describe in more detail the link between practices, trajectories and social structures. Finally, I suggest that various delinquent acts can be linked to three processes whose rationale is *sustaining, producing or overcoming* gender hegemony.

## Masculinities and Femininities

In the now classic *Gender and Power: Society, the Person and Sexual Politics*, Raewyn Connell (1987) establishes masculinities (and, in their mirror image, femininities) as *accomplishments*—“projects” that require acts, discourses, and ideologies. Specific to each individual, “gender projects” encompass the enactment of a gender identity defining everyday practices, language, personal aspirations, and political ideals. Connell posits that masculinities are not essential or static attributes but, as Vigoya (2018: 44) explains, “a historical manifestation, a social construction and a cultural creation whose meanings vary among individuals, societies and eras.” By analyzing masculinities as a “gender project,” Connell (2006: 839) proposes to take into account simultaneously embodied practices, individual trajectories, and what she refers to as “gender regimes”—“the overall pattern of gender relations” within a given institution.

Connell refers to masculinit*ies*, in the plural, because she identifies several, ordered hierarchically. Each of these categories corresponds not to practices or identities *per* se, but to a *place* in the gender order and the *practices* that accompany it (Messerschmidt 2004). Therefore, the attributes and “content” of each masculinity vary situationally (at both societal and institutional levels) and historically. “Hegemonic masculinity” is the culturally glorified form of masculinity that ensures a position of domination over women and over other men.^2^ Other masculinities are then defined in relation to hegemonic masculinity. In this article, I will especially make use of “marginalized masculinity”—a model that does not conform entirely with hegemonic masculinity—due, for example, to the person’s race, ethnicity, class, age, or activities (e.g., ballet, drug dealing, gaming)—but which is still valued in a given group (e.g., in the ballet community, on the street, in the gaming culture or universe). Marginalized masculinities are, therefore, dominated in society and do not challenge the hegemonic model (Connell 1995), but are glorified in certain “marginalized” spaces. Because masculinities depend on context and interactions, men embody multiple masculinities in different life spheres. For instance, in an ethnographic study with Black and Latino boys living in a precarious neighborhood in Oakland, California, Victor Rios (2011) shows how young men enact simultaneously hegemonic masculinity with their peers, subordinate masculinity in school, and hyper-masculinity when facing the criminal justice system. The position of actors in different social spaces makes the embodiment of a particular masculinity possible, relevant, and realistic: a racialized man in a precarious socio-economic position cannot subscribe to the same masculine model as a wealthy, White man—at least not if he hopes to *accomplish* it. Thus, it is crucial to look “beyond gender” in order to grasp the structural context in which it is performed day after day, as it intersects with other power relations such as class, nationality or race.^3^ The entanglement between Connell’s masculinities framework and other structural power relations has been—and still is—debated among scholars (see Hamilton et al. 2019; Henne and Troshynski 2019). In this article, I focus on gender, but I will also discuss interactions with other power relations.

The masculinities framework has been used widely in studies focusing on gender and delinquency. Connell and Messerschmidt (2005), in an article evaluating the framework, drew particular attention to analyses showing how certain crimes are related to the pursuit of masculine hegemony. Studies tend to agree on certain attributes that would define (broadly) hegemonic masculinity among young men who commit crime: competition, heteronormativity, physical strength, sexism and toughness (Abrams et al. 2008; Carlsson 2013; Vuattoux 2018). The literature on “hypermasculinity,” in particular, shows how “the symbolic meanings of hegemonic masculinity are amplified” for marginalized youths (Bengtsson 2016: 414), resulting in situations more conducive to violent, sexual or sexist performances (Abrams et al. 2008). Some qualitative studies, using in-depth interviews and observation, describe how young men (re)produce hegemonic masculinity in detention, how interactions with the police or justice workers are “masculinity tests” or “masculinity challenges,” and how the criminal process enhances hypermasculine traits (Carlsson 2013; Cesaroni and Alvi 2010; Rios 2009, 2011). Some of these studies are exploratory and worth extending; several are based on sources from the criminal justice system (such as judicial records) or are theoretical conceptualizations that would be useful to complement with in-depth interviews. Most importantly, these studies focus on “high profile” offenders, predominantly men, who have committed violent and serious crimes (assault, murder, rape) and been incarcerated. Yet, the strength of the framework as developed by Connell rests in the articulation between different layers of hegemony—including when there is an absence of hegemony. It is thus important that masculinity *and* femininity, hegemonic *and* non-hegemonic positions be studied together, in a holistic approach, to understand the “gender regime.”

According to Connell (1987: 183), “there is no femininity that is hegemonic in the sense that the dominant form of masculinity is hegemonic among men.… It is the global subordination of women to men that provides an essential basis for differentiation.” There is, of course, “the most dominant among the dominated” (what Connell (1987: 183) refers to as “emphasized femininity,” which counterpoints masculine hegemony: a woman who is “soft, submissive, sexually coy, alluring or flirtatious, concerned with domesticity and preoccupied with bodily appearance” (Bradley 2007: 48). Following Connell’s seminal work, some feminist scholars began to take an interest in “de-homogenizing” this conception of femininity. “New femininities” have emerged as an area of inquiry, which interrogate the (potential) transformations in models of femininity due to second wave feminism, new technologies, globalization and neoliberalism (Gill and Scharff 2011). None, however, have matched “the focused study of masculinity…nor has there been a sustained attempt to investigate ‘hegemonic femininity’” (Budgeon 2014: 321).^4^

Mimi Schippers is one of the few to have reworked Connell’s model with the aim of integrating multiple femininities. According to Schippers (2007: 94), we should focus on “the idealized relationship between masculinity and femininity”—which is based on a complementarity and a hierarchy between the sexes. As an additional category in the model, she proposes “pariah femininity,” which encompasses women who “embody and practice” masculine traits (for instance, sexual, attraction to other women, authoritarian or aggressive behavior) (Schippers 2007: 95). By adopting masculine practices, and thereby crossing a gender border, women *challenge* masculine hegemony. This femininity is, therefore, not only subordinate or marginalized, but “pariah” because it refuses to complement masculine hegemony and disrupts the gender “equilibrium.” In summary, Schippers proposes to analyze femininities and masculinities *through their relation to the *“gender regime,” by asking which masculinities and femininities comply with, or disrupt, the established gender hierarchy? Focusing on delinquency raises a number of questions: How do masculinities and femininities translate into delinquent practices and norms? Where do youths—boys and girls—who have committed offenses fit in the “gender regime” hierarchy?

## Background and Methods

The data presented in this article are drawn from an ongoing sociological team research study, based on a multi-sited ethnography, to analyze the experiences and trajectories of young people in the Swiss juvenile justice system.^5^ The Swiss legal framework grants priority to treating minors as subjects in need of individualized assistance. Under the Swiss Juvenile Criminal Law Act (*Droit Pénal des Mineurs* (“DPMin”)), revised in 2007, judges are expected to focus on the youth’s “living conditions and family environment,” as well as on the “development of their personality,” rather than on the offense. In order to implement this person-centered justice, sentencing is carried out within a dualistic framework. Like those of other European countries, the DPMin distinguishes between protection measures—which have a therapeutic or educative goal, such as social monitoring (*assistance personnelle*) or community home placement (*placement*)—and sentences, such as detention (*privation de liberté*) or community service (*prestation personnelle*), which are intended to punish, if the minor had acted “in a knowingly guilty way.” Pursuant to the 2007 revised DPMin, sentences and protection measures can be combined to serve both retributive and educative objectives^6^; young people, as I describe below, regularly experience both measures and sentences simultaneously.

Two additional points are necessary in order to understand the Swiss context regarding youth delinquency. First, gang-related issues and juvenile crime organizations are absent from the public and judicial debate. Rather, youth delinquency is officially framed as an individualistic problem related to familial dysfunction, school dropout, or psychosocial issues (Zermatten 2007). Second, the available statistics on juvenile delinquency do not specify age, class, race or sex.^7^ In other words, a demographic analysis of juvenile delinquency is virtually impossible in Switzerland because the aggregated numbers obscure the role of social determinants.

These two points are consubstantial with the system’s principle of individualization, which is ultimately a double-edged sword. This principle allows a personalized response to the situation of each youth but, in so doing, it contributes to the reproduction of inequalities (based on class, gender, race), as sociologists have long recognized (Chamboredon 1971). Table 1 contains basic demographic information about the study participants to date, distributed between four penal trajectories according to the sentences the youths received. Although the study is not representative, these demographics show that racialized youths, youths who come from a precarious background, or those who have dropped out of school, are over-represented. This demonstrates that the individualization principle does not allow structural inequalities to be addressed better.

**Table 1:** Participants’ demographics

Penal trajectories	Participants	Sex		Ethnicity/race		Swiss citizenship		Education^a^				Class (self-assessed)		
		Male	Female	White	Racialized minority	Yes	No	Drop-out	Mandatory school	Vocational school	High school	Very precarious	Working class	Middle class
Open custody	8	6	2	3	5	5	3	1	3	3	1	3	4	1
Secure custody	13	10	3	6	7	8	5	7	2	4	0	7	1	5
Mixed	8	6	2	4	4	7	1	0	2	5	1	5	1	2
Single offense	5	2	3	4	1	4	1	0	0	2	3	0	2	3
Total	34	24	10	17	17	24	10	8	7	14	5	15	8	11

^a^Switzerland has a dual-track educational system that separates young people (approximately 16 years old) between an academic track (high school) and a vocational track (apprenticeship)

This “individualization setting” provides a rich ground for analyzing how hegemonic structures reproduce themselves through individuals on a wide range of paths. The research project was thus designed to apprehend the varied trajectories of young people within the justice system. We contacted several institutions across the French-speaking part of Switzerland and negotiated with them to meet young people. Eventually, eight institutions put us in touch with youths who were in their care at that time, of whom we met thirty-four.

We chose as a selection criterion “having met a juvenile judge at least once.” In doing so, we wanted to expand the research of previous ethnographies (Marcus 1995) and encounter all possible trajectories in the penal system by meeting youth who had a very brief encounter with the justice system, as opposed to only incarcerated youths, and by engaging with the groups that tend to “disappear” as the severity of the sentence increases, such as girls or youths from wealthy families (Guevara et al. 2006; Herpin 1977; Vuattoux 2014). As a result, our corpus contains quite a broad range of penal trajectories—in terms of length, frequency and type of sentences. Table 2 presents examples of the four types of penal trajectories that were encountered in the study. The eight interviewees depicted below were chosen for their differences—illustrative of the diversity of experiences with the justice system.

**Table 2:**
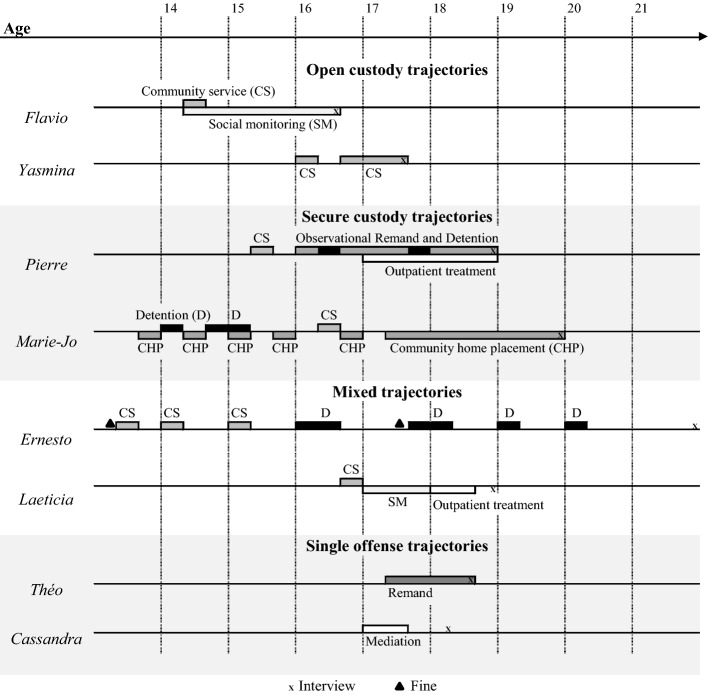
Types of penal trajectories in the swiss juvenile penal chain

As part of our ongoing research project, we conduct life history interviews with the youths and engage in ethnographic data collection in judicial institutions (e.g., observations, informal and formal interviews with judges, social workers, and other criminal justice system actors). In general, the life history interviews last between one and a half and three hours, and the context in which we meet youths depends on their specific situations: in an institution for those in foster care or detention, otherwise in a place of their choice, like a café or park. We start each interview by asking the interviewee to recount the broad lines of his or her path in the justice system. Using this framework and depending on the trajectories described, we then explore such themes as deviant and delinquent trajectories, family life course, institutional and judicial paths, professional career and schooling. The youth’s participation is voluntary and we take care to insist on several points at the beginning of the interviews: information provided to us will not be transmitted to or shared with anyone else, especially with those working in the justice system; meeting with us will not affect (either positively or negatively) the youth’s trial or sentence; at any point, the youth can decide not to answer a question or to stop the interview. Finally, we indicate that all information will be subjected to an anonymization process (modifying names, dates, and sometimes offenses, if these make interviewees too recognizable) before being communicated publicly or published, as is the case in the present article. We have also decided against collecting information on the trajectories or offenses of the youth before meeting them; the study includes youths who are currently under investigation and thus often have to face justice and social work professionals in a setting identical to ours—namely, being questioned by an adult. Not knowing the delinquent past of the interviewees would, we reckon, place us in a better position from which to negotiate the “trust agreement” in the interaction.^8^

### Analytical Strategy

I undertook analysis of the data using the software Atlas.ti and a “flexible coding” approach (Deterding and Waters 2021). First, I coded the transcribed data (life history interviews with youths, informational interviews, fieldnotes) using a descriptive and open coding strategy. This allowed me to identify, among others, the four types of penal trajectories presented above and to note prominent topics. I then approached the life history interviews with a theory-driven coding strategy, focusing on masculinities and femininities. This second coding, once structured in *code families*, provided an outline of relevant topics across the interviews.^9^ By using the *query tool* and *co-occurrence tables*, I was then able to look for connections between topics, individual trajectories, and social characteristics.

The analysis presented here is based on the entire fieldwork (interviews, observations). In order to highlight how masculinities and femininities are embodied in a specific trajectory, I first present in detail the trajectories of Ernesto and Yasmina. This focus enables me to reveal how a single individual enacts multiple masculinities or femininities. I have selected Ernesto’s and Yasmina’s cases because these two interviews are remarkably rich regarding gender relations and practices, and are illustrative in terms of multiple masculinities or femininities embodiment. Moreover, their similar social situations allows a clear comparison from a gender perspective. After analyzing these two trajectories, I qualify them by making reference to other youths whose situations are less sharply polarized, and show how practices, trajectories and social structures interact with delinquency.

## Delinquent Trajectories and “Gender Projects”

### Ernesto

You can’t wait to have worked a month to get paid. No: my little sister’s birthday is in two weeks, so I need this money in two weeks to buy her “that.” Because she told me she wanted “that” …. I wanna be proud of myself, and I want my family to be proud of me. I can’t get that by working, so I’ll get it some other way.^10^

Ernesto recounted his delinquent trajectory to me while we sat in a coffee shop with his best friend. At the time of the interview, he was twenty-one-years old, and had desisted from crime for about a year. Ernesto explained to me his past motives for his delinquent behavior. These were primarily economic, geared to funding “leisure” (clothes, parties, trips), but were also undertaken to support his family that lives in extremely difficult conditions. Ernesto described himself as a “teen version” of the male breadwinner. This image is one that often occurs in interviews with male youths: they want to “help mom” to “pay the bills.” In other words, they aspire to relieve their parents—mostly single mothers—of financial pressures that they consider shameful. Delinquency presents some opportunity for individual initiative to lives that are often characterized by numerous obligations.

Ernesto was clearly marginalized in relation to Swiss society: along with his nine brothers and sisters, he was brought up in a working-class area by a single mother with health issues; later, he dropped out of school. Today, “thanks to [his] social worker,” he has a qualification in the building trades, but he struggles to find work and is not satisfied with his employment. To this day, Ernesto’s financial contributions remain central to the family budget—a responsibility he shares with the friends with whom he started committing crime: “We all grew up together in my neighborhood. … We see our mother crying at home, we see the bills piling up, we see the empty fridge, we see… we need money.” In this statement, as in others, the repeated use of “we” shows Ernesto’s strong sense of belonging to a group—that of young men struggling at home and at work, who have so far spent their lives “fighting” the police, the justice system, and the job market. To use the terms popularized by Richard Hoggart (1957: 62–68), Ernesto expresses a conflict between “Them” (“the higher-ups” that will “get yer in the end”) and “Us” (“We are all in the same boat”).

In short, Ernesto explained his offending as a way to acquire the resources he and his family lacked. Pursuit of these resources had led him to commit a series of offenses that were serious and exceptional compared to those of the other youths in the study: he had been charged with break-ins, burglaries, drug trafficking, human trafficking (for the purpose of prostitution) and, most recently, multiple group fights. In the course of his penal trajectory, he had accumulated numerous hours of community service, spent time in prison, and had been sanctioned by social monitoring.

The money he had acquired from his illegal activities was used to pay for family expenses—what he calls “needs”—but also for certain “extras … that are also needs, in a way”:

The most memorable was that vacation in Copenhagen, I went away thanks to that [the money acquired through his criminal activities]. On Friday, we [Ernesto and his friends] did what we needed to do and the day after we bought our plane tickets. It was summer, everyone was going on vacation. And we weren’t! We didn’t have any money… how could we go on vacation? Then a friend says: “Come on, we’ll make some money, come on, let’s go on vacation.” And that’s exactly what we did … In one week, I blew twenty grand. Twenty grand! I can tell you something: we did Copenhagen all right. Ah, makes you feel good, right. Booze, hookers, you name it.

This vacation and the forms of resources it mobilized, provides a fine illustration of how delinquency is related to “gender projects” and the pursuit of hegemony.

First, several attributes or features associated with “classic” Western hegemonic masculinity, which became available to Ernesto through delinquency, are present here: freedom, hedonistic pleasures, heteronormativity, money. Second, precisely because of “big hits,” like the one he performed before the Copenhagen trip, Ernesto gains “street cred” and is recognized by people in the streets.^11^ In his social world, his “turf”—which he compares to “a housing project in France, any ghetto you can think of”—he is a public figure. He is perceived as “a real professional” with his “criminal specialty”—one that he refuses to reveal—and is noted for his relational skills in the criminal world. Strangers interested in doing “profitable business” still accost him, even though he “retired” over a year ago.

When Ernesto was still engaged in a delinquent career—he is now trying to “stay on the rails*”*—he thus embodied several masculinities: a hegemonic masculinity in his subculture, where he possesses symbolic and economic resources, based on his criminal skills and physical skills, derived from being a local karate champion. Here, he is a “tough guy” and has status. In addition, being involved in delinquency puts him in a powerful position. For his peers, he symbolizes strength and intelligence—when he is not caught—or courage and resistance, when he gets sent to prison. Thus, delinquency not only provides him with the resources to “act” in a hegemonic way, but it becomes a *hegemonic marker*, in itself. Yet, this hegemony is strictly context-bound, for when viewed in a broader perspective, Ernesto’s economic resources were acquired through risky and illegal activities, his agonistic resources are worthless in respect of legal or social institutions, and his status does not enable him to look to the future with optimism, at least not concerning the legal labor market. In contrast to his “delinquent” identity, which is relatively hegemonic, Ernesto’s broader situation bears all the hallmarks of *marginalization*.

This apparent paradox emerges clearly in Reich’s study (2010) on juvenile offenders in prison, who are simultaneously glorified in pop culture *and* socially marginalized. It would be difficult “to show *either* that these men enacted hegemonic masculinity, given their subordinate economic and political positions¸ *or* that this kind of masculinity was subordinate to others, given its cultural prominence and power” (Reich 2010: 15–16 (emphasis in original)). This ambivalence, as well as the offenses committed by Ernesto, fits particularly well into the hypermasculinity framework mentioned earlier: the more marginalized young men are, the more they perform an exaggerated version of hegemonic masculinity. Thus, in Ernesto’s trajectory, we observe an extreme form of domination—over men (as described above) and over women (e.g., by paying for sex, by participating in the trafficking of women).

### Yasmina

First, I have a problem with authority, and especially when it’s masculine. So I think it comes from my father, I mean, it’s something I’ve noticed. I really struggle with masculine authority. … Anyway, it looks like I screwed up…Yeah. I needed that to change course and … grow up, in a way.

This interview excerpt illustrates how Yasmina, who had just turned eighteen when we met, apprehends her delinquent trajectory through the prism of psychology or personal development. Whereas masculinity is seen as “intrinsically” linked to delinquency and violence (Dowd 2008), no logical link has been acknowledged between crime and femininity by either researchers or criminal justice system actors (Cardi 2007). In contrast to most boys who omitted these aspects in the interviews, girls explain their criminal behavior as the product of relational, psychological or emotional instability. Having had lengthy contact with criminal justice and social institutions, Yasmina believes that the reasons justifying her “screw-ups” are not external but internal, individual, and emotional. Her family environment, however, is similar to Ernesto’s: she was raised by a single mother (later remarried) who has health and addiction problems and for whom money is a constant worry. When her parents divorced—Yasmina was then two-years-old—child protection services were alerted to the family’s highly contentious situation. Thereafter, Yasmina was never off the radar of the social services network. From early on, her schooling became difficult, marked by repeated instances of dropping out and multiple expulsions. Yasmina’s file contains two offenses: (1) a minor theft in a supermarket; and (2) an act of physical and verbal violence against one of her teachers, who was also the school’s dean and who lodged an official complaint. Yasmina was banned from the supermarket for a year, without being sent before a judge. For the second offense, however, she was ordered to appear in court and sentenced to community service in a nursing home and anger management training in a specialized institution. On the whole**,** however, Yasmina’s contact with the justice system has come mainly through the civil courts, in a non-criminal context: she has run away from home many times and has a difficult relationship with her mother, mostly due to the latter’s addiction problems. She has spent several years in a youth care facility and has been enrolled in several socio-professional integration seminars^12^—all of which adds up to a chaotic trajectory that Yasmina has internalized: in her narrative, the lines between criminal justice and social surveillance are inevitably blurred.

Like the other girls I interviewed, Yasmina does not see a “collective” dimension to her actions: she uses only “I”—never “we”—and is not interested in bonding with other girls or boys who are experiencing similar situations. In sum, she thinks and reasons in “Me” against “Them” terms. One possible explanation for this difference form boys’ narratives is the process of medicalization/pathologization Yasmina has experienced, which identifies individual mental instability as the source of (her) delinquency (Vuattoux 2014). This process is strongly gender-biased (Amsellem-Mainguy and Dumollard 2015) and could explain, in part, these two distinct gendered narratives.^13^ Moreover, in terms of gender roles and ideals, Yasmina does not see herself as part of a larger group of girls:

Yasmina: My “girl” buddies, they’re just like me. My “girl” buddies do not like girls, the same as I don’t like girls.
Interviewer: You don’t like girls?
Yasmina: The girly-girls, you know, the [in a high-pitched voice] “oh no, I’m not speaking to you because of this or that” kind of girls. No. I mean, you’re not gonna die if you break a nail, sweetie, relax!”

A common feature among the young female interviewees is their readiness to criticize what I previously referred to as “dominant femininity” or, at least, its teenager version: the “affected,” “bitchy” girls; the “precious little things,” who like to stay “just us girls” and who “are always trying to attract the boys” and who, in contrast to the interviewees, are not seeking empowerment and self-determination. Not all types of femininity or feminine characteristics are rejected, however: Yasmina, for instance, who describes herself as a “tomboy” (“I swear, I’m not afraid to be violent, impulsive”), behaves in specific contexts like a “real woman” (“I wear heels and make-up…I’m shy, I talk about my feelings”). The femininity that she enacts thus comprises an ongoing negotiation between a traditional position, subordinated to masculinity, and a “pariah femininity,” in search of autonomy and power. She deploys relational, emotional and aesthetic resources that typify what she identifies as feminine, but also resources that have emphatically masculine associations, such as independence, toughness and a willingness to engage in physical and verbal violence. Indeed, she rejects friendships with “girly-girls who always get worked up and are two-faced” and who she thinks make up the majority of girls in her age group. In this respect, what started the following situation, referred to by Yasmina as “the dean incident,” is revealing:

So yeah, a girl from the school kissed him [her boyfriend], and you could say I just flipped … [When she went to the school to confront the girl she asked around “where is this slutty Maria of yours?”] Actually, she’d gone to hide in the dean’s office! Just to show you what a wuss she is—anyway, I come up to her and she starts going “No, it’s not true, I didn’t kiss him, he kissed me,” I said “Bullshit, what are you making up, everybody told me and everybody saw it.” [Her boyfriend and the dean arrived, the other girl hid. An argument started between the dean and her boyfriend who began to act violently; the dean called the police to take him away. Yasmina went outside, smoked a joint, and then returned to talk to the dean.] He’s like “oh, so you’re Yasmina, you came here to mess with Maria, you wanna act tough”, etc.… There—I said something, it just came out—I said “son of a bitch.” He says to me “really, you know my mother?,” and I’m like “sure, she’s my dad’s bitch” [pauses, laughs]. He’s like “ok, fine, get out of this establishment young lady,” I start walking away and I turn round and say “you can all tell this little slut I’ll cut her head off tonight.” “Of course [laughs], of course they filed a complaint against me for death threats and insults, there you go .…

Two gendered mechanisms can be identified in this episode and its narrative construction. First, the constant efforts Yasmina makes to play the dominant role—whether with the dean, the other students, her boyfriend, or with me during the interview—are striking. Her behavior is at odds with all the traditional feminine attributes: she is neither soft nor submissive; she uses crude language; and she acts as if she does not care about the consequences. Second, in sharp contrast to her everyday persona that is, to a large extent, controlled by institutions, this offense allows her to “act” in a hegemonic way by rejecting all authority. Just as in Ernesto’s case, this action becomes a hegemonic marker in its own right—by increasing her “reputation” and her symbolic dominance amongst other teenagers. In other words, where Maria is “a wuss,” “a liar,” “a snitch,” and “a slut,” this episode gives Yasmina the opportunity to demonstrate that she is none of these things.

## In Search of Hegemony: Time and Social Structures

One important feature of Connell’s framework is that masculinity and femininity projects encompass individual practices and trajectories, as well as social structures and temporal configurations. So far, by focusing on two respondents, I have described how delinquent practices and trajectories are linked to the “gender regime.” In this part, drawing on interviews with multiple young people, I explore how “gender projects” and delinquency interact with structures and configurations by posing the following questions: (1) How do “gender projects” change with the passing of time? (2) Who is able to commit offenses without coming to the attention of the justice system? (3) For whom can delinquency be a way of acquiring hegemony? In other words, can every youth benefit from delinquency in the same way?

### The Future is Gendered: Masculine and Feminine Models of Adulthood

“What it means to do masculinity changes with age” and, as we have seen in the case of Ernesto, as time goes by, committing a criminal offense “is no longer compatible with the desirable masculine status” (Carlsson 2013: 675). The gap between risky practices and deviant lifestyles, on the one hand, and a hegemonic model defined by economic power and status, on the other, is present in the discourse of most young men. A point that emerged from analysis of the interviews was the tendency for this disparity to become harder to accept with the passing of time. In addition, the ideal model presented earlier—centered on freedom, money and power—tends to be replaced by a much more conservative one.

This ideal model corresponds to an overwhelming desire “to settle down.” First and foremost, in relation to delinquency, the young men want to “redeem” themselves, “go straight,” and earn respectability. Then, in other life spheres, they want to: “Have … little kids, find a real woman, have a kid perhaps, get married, find a job” (Ernesto), “do something with my life” (Andre), and, especially, “to owe nothing to anyone” (Felipe). Indeed, while most of the young men interviewed enact a delinquent masculinity in which physical and economic power serve to procure a hedonistic lifestyle—with adventure and kicks, status and reputation—they all aspire “to settle down” as early as possible: it all comes down to taking responsibility.

In *In Search of Respect: Selling Crack in El Barrio*, Philippe Bourgois (1995) provides a fine account of this shift in outlook among drug dealers in “El Barrio*,*” a New York neighborhood. Bourgois shows that while independence and autonomy are central goals for his respondents, the legal labor market offers them only a low and subordinate status. Two possibilities are open to them: succeed in delinquency, which carries a risk for their autonomy (if they are apprehended), or accept a dependent situation in the labor market, which ensures a relatively quiet life. The choice of one path or the other depends primarily on acquired resources or opportunities, and desistance studies show us that in the vast majority of cases, “going straight” is a long and difficult process with many setbacks along the way (Maruna 2001; Rios 2009).

Another attractive status for some young men is the “wounded healer narrative” (Maruna 2001: 117), which involves re-crafting “a delinquent history into a source of wisdom to be drawn from while acting as a drug counselor, youth worker, community volunteer.” The young men want to serve as *examples*—or, more accurately, as *counter-examples*—for their younger siblings or for the “little brothers” of their neighborhood, and not simply in their professional ambitions. Flavio, a young dealer, recounts some “triggers” from when he started to scale back his delinquent activities:

Not being a bad example for my brother, changing my future. Thanks to this, actually, my buddies… two or three buddies of mine copied me, they said: “yeah, you know, we’re gonna do it your way. … they came to me and said: “Fuck, Flavio, you really opened my eyes”. Makes you feel good, y’know, ‘cos I managed to get them out of a bad situation.

The idealized masculine future (“settling down”) is thus linked to long-term familial and financial security—and being “exemplary.” On the girls’ side, emancipation from institutions of social control (e.g., family, parents, social workers) is their major goal for the future, albeit an individual and short-term project. The embodiment of an autonomous gender role and the pursuit of agency in situations marked by its absence are common to all the girls that I met. Most young women declare that the quest for autonomy and agency does not end with age or desistance processes. As twenty-six-year-old Apou puts it, “I didn’t know, then, why I was dealing drugs, y’know? But for real, I just wanted to be left alone, live my life as I wanted to. And nowadays, it’s exactly the same thing.” As such, girls’ gender ideals and the criteria that guide their “gender projects” seem broadly similar to those guiding the boys’ projects. Taking on responsibilities and achieving independence—emotional, financial, and professional—is central to the young women’s concern. Rather than negotiating gender hegemony by settling in society, as was the case for young men, the young women I interviewed seek to extricate themselves from it. I posit that this divergence is due to the lack of opportunities for young women to gain hegemony in the patriarchal “gender regime.” Indeed, finding a husband, having children and a casual job (the feminine version of “settling down”) would place them, again, in a subordinated situation. The female respondents dream of finding a new home, of moving away from their family and from “child status,” and of having a job they are passionate about (e.g., baker, sign language interpreter, tattoo artist); they aspire to “choose my own way” (Yasmina), “to be left alone and get on with my life” (Apou), and believe that “it’s up to me to change my future” (Ana).

As Irwin and Chesney-Lind (2008: 847) put it, “we must understand gender as a fixed component of a sex/gender system that organizes every context in which girls and boys grow up. In addition, we must acknowledge that gender is also flexible, negotiated, and achieved variously in different contexts, as West and Zimmerman articulate.” Coming from a marginalized, unprivileged family, and having no academic or professional qualifications, Yasmina—like the other as female respondents—embodies, expresses and nurtures her desires for autonomy in a specific way: outside of the traditional gender role patriarchy has assigned her. Through young women’s delinquent acts, we can thus observe the rejection of gender roles that girls consider unacceptable and the concomitant search for female hegemony.

### The Social Space of “Gender Projects”

If we move away from gender as a distinction criterion, the individuals in the cases that I have discussed share a specific social situation: both Ernesto and Yasmina, as well as the youth mentioned in the previous part, dropped out of school, were raised by working-class single mothers, and are second-generation immigrants. But the multiple realities of juvenile delinquency are more nuanced than this: some of the young people interviewed have professional or educational goals, come from upper-middle class backgrounds, and have satisfying forms of sociability inside and outside their families. Furthermore, juvenile delinquency studies, especially those based on self-administered surveys, suggest that the majority of young people break the law at some point, but “eventually move away from it” (Benazeth et al. 2016: 324). It is certainly fair to assume that young people of all classes, nationalities and races commit criminal offenses, although only some of them are brought to justice.

This could mean that not all youths have the same “ability” to hide their delinquent acts—to deviate from laws and norms without being sanctioned. This hypothesis is supported by numerous studies that show disparities in police encounters and in judicial and extra-judicial responses, depending on class, gender, and race (Bradford 2017; Skogan 2017). Some respondents in the present study conclude that the reason for their frequent interactions with the criminal justice system lies partly in the position they occupy in the social space:

I said to myself: yeah, what about if, right now, I was a hard-working forty-year old? And if I had the same problems that I’m having today, would they treat me the same way? Nah, no way. They would never treat me the same. (Roby)

Really, when you’re a colored person… Well, we get controlled more often by the police, like, for real. It’s—I’ve seen it, witnessed it, experienced it! And the way they talk to us, most of them, they don’t talk that way to other people that is… you know? (Flavio)

Some professionals confirm these biases. For instance, when I questioned a judge about the background of the youths who come before him, he replied that upper-class Swiss youth rarely appear in “his court”—not because they never break the law, but because they tend to commit minor offenses that do not involve going to court and are settled with a small fine^14^ or because the youth’s “living standards, home environment and personality” are deemed “healthy.”^15^ These evaluations that rely on the individualization principle mentioned above—which will determine the youths’ path in the criminal chain—cast light on the structural mechanisms of delinquent trajectories. As a function of social determinants (age, class, gender, nationality/origin, race) and the related resources—like articulacy (Bugnon 2017) or self-presentation skills (Werth 2012)—the young people may (or may not) remain unnoticed and therefore achieve their “gender projects” through delinquency.

The above constitutes a first attempt to take into account social structures and power relations other than gender. A second way to consider power structures is to contemplate whether individuals have equal opportunities when it comes to engaging in delinquency and benefitting from it. From the respondents’ point of view, delinquency constitutes a means to an end (autonomy, emancipation, financial gain and independence) and sometimes an end in itself (improved status within their group, providing agonistic, reputational and statutory resources).^16^ All are indicators of hegemonic status or characteristics that are universally desired—by deviants and non-deviants alike. Delinquency aside, it is striking that all of these aspirations are close to “normatively dominant values.” In other words, what Ernesto, Yasmina, and the other young people I interviewed are looking for does not make the case for “delinquent values” but actually validates “mainstream values” (Matza and Sykes 1961). If the girls resemble the boys that were interviewed in trying to use delinquency to acquire hegemony, however, or at least certain components of hegemony, we saw that the means deployed to achieve their “gender projects” vary: if “doing gender” is different for men and women, they also “do crime” differently (Miller 2014). The hypothesis I wish to consider, then, is that while delinquency can provide hegemonic resources for young people, the acquisition of such resources (or not, as the case may be) depends on an individual’s position in social space.

Contrasting sharply with Ernesto in terms of background is Nathan, a White middle-class Swiss boy I interviewed who embodies a hegemonic masculinity. He is passionate about his part-time job as an illustrator, serves as a role model for his “mentees,” is popular among girls of his age, and has financial security. One night, he took part in a group assault with his friends, which received considerable media attention. Nathan says he does not “really know why” he participated, especially because he does not consider himself a violent person. Two non-exclusive hypotheses can be made: first, taking part in the assault was a way to produce a marginalized identity that met the expectations of his friends, most of whom are working-class teens “used to street fights.” Second, the offense sustained the gender hegemony he currently embodies by reasserting his status and strength, his courage, and his solidarity with his friends. Because the attack was particularly serious, Nathan was pursued and arrested by the police, though he says he was “treated correctly and fairly.” Nathan made no attempt to produce a different gender identity while in custody. Instead, he was cooperative, aware that a hostile attitude would be counterproductive if he wanted to make his time in the criminal justice system as short as possible: “If you are difficult, if you try to be . . . try to be the tough guy, the delinquent, like ‘I don’t care, don’t care a bit, I’m not saying a word’ it won’t do you any good….”

Turning now to the girls’ side, I interviewed Cassandra, who has professional training and a job and who also engages in voluntary activities. Although not from a wealthy family, she has financial security and her parents have invested a lot into her future. On one Christmas Eve, Cassandra was caught on camera pushing a woman when a fight started between her boyfriend and other customers at a restaurant. Cassandra was very cooperative with the police and even has a positive memory of her encounter at the police station, saying the inspectors “were really nice and understanding.” Like Nathan, she does not “really understand what happened.” It becomes clear during the interview that Cassandra seeks to embody a traditional form of femininity: she considers herself to be a “good girl,” who does not like violence but who stands by her boyfriend when he “messes around with his friends.” She also expresses her appreciation for the complementary (and hierarchical) relationship between masculinity and femininity: girls are “*supposed to* advise boys not to mess around . . . and take care of them afterwards, because they don’t listen.” Within our analytical framework, we can interpret her offense as a way to consolidate this “traditional” position in the gender order, while at the same time, reinforcing that of her boyfriend. Compared to Yasmina, who has few economic and familial resources and is seen as a “troubled teen” who needs “bringing into line,” Cassandra is able to “handle the situation.” Following the hypothesis formulated at the start of this section, I can conclude that Cassandra’s social characteristics enable her to benefit from her violent performance. She views her offense and the resulting interactions as “a bad memory . . . something that happened, one more thing in my life, but that’s all.”

## Sustaining, Producing or Overcoming Hegemony

Youth of both sexes are therefore related to gender hegemony in various ways, through both their delinquent activity and through their broader social positioning. Eschewing social reaction theory (according to which girls and boys “do crime” differently because they are labeled and treated differently), I have tried to show how boys and girls realize different “gender projects” through delinquency. By looking at other power structures, I have suggested that not every young person can benefit from offending in the same way, if at all. Some teenagers possess certain hegemonic characteristics and “work hard” to maintain them (Cassandra and Nathan); others stand in a marginalized position and try to produce hegemonic features in parallel sub-worlds. Among the latter, some do not challenge the gender order (Ernesto), while others defy it (Yasmina). Schippers’ (2007) conceptualization of hegemony as a relational entity does, therefore, contribute to our understanding of juvenile delinquency. By taking into account masculinities and femininities, and their relationship to hegemony, as well as social space positioning, I can identify several processes that seem to coexist. 17 Some youths engage in crime to *sustain* hegemony, others do so to *produce* a “marginalized” hegemony that does not conflict with the “gender regime” and still others finally seek to *overcome* hegemony, by rejecting the “gender regime.” Evidence from this study shows that the last process, overcoming hegemony, is enacted only by women. This could suggest that young women have no choice but to reject the patriarchal “gender regime” in order to gain self-determination, whereas young men, even in marginalized situations, can find some form of power by reproducing it.

In addition, however, we saw that committing crime does not expose everyone to the same type or level of risk. It is easier (or less risky) to sustain a hegemonic position than to overcome a subordinated status. Except when a particularly serious offense is committed, as in the case of Nathan, White upper-class teenagers in Switzerland seem relatively invisible to the criminal justice system. Thus, it is important to stress one last feature related to gender hegemonies: the majority of young people deflect legal norms to some extent, in that “‘abnormal’ teenage behavior is just a ‘normal’ part of growing up” (Slater 2016: 2). Abnormality—in the present context, delinquency—has a cost that not all young people are able to pay, and the “ability to deviate” is not distributed equally (Slater 2016: 15–16). Being unpunished or unlabeled but also being able to support the costs of a potential criminal trajectory becomes an additional aspect of hegemony.

## Conclusion

According to Steffensmeier and Allan (1996: 459), “criminologists agree that the gender gap in crime is universal.” To date, research has addressed the issue in different ways, but rarely from the perspective of young people. While Connell’s masculinities framework has proven to be a fruitful way to expand our knowledge of this issue, the theoretical concepts of “hegemonic masculinities” or “pariah femininities” need to be subjected to an in-depth analysis of lived experiences in order to contribute to the debate as well as to social change (Carlsson 2013). Looking at a gender hegemony framework for young people provides us with valuable insights because it allows us to analyze how patriarchal structures influence and reproduce themselves in the lives of boys and girls. Based on a qualitative analysis of interviews with youths, this article has shown how a plurality of masculinity and femininity models coexist for these youths. These models are associated with a variety of delinquent practices, gender identities, rationalizations, and social positioning. While the resources pursued through crime by boys and girls are ultimately similar, how these resources are used vary. In sum, the observations presented bring out the fact that juvenile delinquency serves a profoundly normative project. The “values” or “goals” underpinning the cases I have analyzed are autonomy, financial independence, freedom, and relational power. These values are not only accepted but—in Western societies, at least—actually *encouraged*. This study also suggests that multiple power structures need to be taken into account when analyzing young people’s delinquent acts and “gender projects.” Patriarchy has been the focus of analysis of this article, yet my study suggests that patriarchy interfaces with class and race structures in a specific way regarding delinquency, now making it impossible for some young people to benefit at all from delinquent acts. Exploring in more depth how affluent White youths experience delinquency and negotiate gender hegemony is therefore necessary for tackling class and race stereotypes, which ultimately affect labeling and sentencing.

The heterogeneity of young people’s experiences reflects the multiplicity of social positioning and relationships to hegemony: offenses serve gendered projects to acquire, maintain or defy hegemony. Because gender roles remain binary and rooted in a patriarchal regime, the ways of interacting with hegemony are compartmentalized, and adolescents do not benefit from delinquency in the same way. This is not to say that crime is related *only* to gender hegemony, or that *all* offenses serve normative goals; rather, the mechanism at work here is intertwined deeply with labeling processes, and with gendered socialization and interactions. Crime can be explored as a set of practices that reveals ideals, identities, and the internalization of hegemonic norms.

## Footnotes

1 By “domestic environments of young people,” I mean group home placement, emotional and familial context, or schooling (Bibard et al. 2016).

2 “Hegemonic masculinity” has been be typified, for example, as “the strong, technically competent, authoritative leader who is sexually potent and attractive, has a family, and has his emotions under control” (Acker 1990: 153). “Hegemonic masculinity” need not have any actual individual embodiment in society. It is symbolic and localized, and it can exist solely in the collective imagination (for instance, superheroes in movies or in literature).

3 In its initial formulation, “intersectionality” (or “interlocking systems of oppression”) aims to make visible and to articulate the multiple oppressions faced by Black women (Combahee river collective [1977]1986; Crenshaw 1989). Living complex lives subjected to sexism, racism and classism, these women fought for a more holistic approach to gender (and feminism) and for recognition of the intertwining discriminations they faced.

4 Moreover, Budgeon (2014: 321) highlights that “gender research has become more attuned to a multiplicity of femininities” but “it is not entirely clear” whether they affect the gender order.

5 “Youth facing the Justice System. Analysis of the criminal chain through the experiences and trajectories of juvenile offenders.” Project funded by the Swiss National Science Foundation (1st Division, Arnaud Frauenfelder, Franz Schultheis, Géraldine Bugnon and Armelle Weil).

6 The country’s legal approach to juvenile delinquency is broadly comparable to that of most European countries, positioning itself as a “moderate educational and justice approach” system (Dünkel 2014: 60).

7 On the specific issue of racialization, Switzerland bears the marks of “raceless Euroracism” and the public debate about race is very different from that in Anglo-Saxon countries (Michel 2015). The widely shared image of Switzerland emphasizes its “non-possession of formal colonies, neglects the country’s multiple entanglements within transnational colonial projects, and thus conceives ‘Swissness’ as external to (post-) colonial history and racism” (Michel 2015: 410).

8 This is one of the strategies we deployed in order to create a safe environment during the interviews. Our method of recruitment and the particular situation of the interviewees of this study, however, has involved overcoming important challenges (see Weil et al. 2021). Mainly, we had to navigate both criminal justice actors and youths, and to manage this tricky intermediate position. First, we needed to earn the trust of criminal justice actors in order to gain “access” to youths. That stage proved to be difficult—in part, because our work as researchers did not always coalesce with the intervention goals of judges and social workers. Second, we had to convince youths that we were genuinely interested in their perspectives, and that we were not collaborating with the justice system, even though we met them via criminal justice actors.

9 These topics are, *inter alia*, gendered practices and attributes (codes: “competitive practices,” “bodily appearance,””physical power,” “care”), discourses about gender and views on the gender hierarchy (codes: “gender complementarity discourse,” “girls don’t do crime,” “gender subversion discourse”), and self-positioning within the gender hierarchy (codes: “tough guy,” “not a girly girl,” “respectful girl/boy”).

10 All quoted excerpts are translations of interviews conducted in French.

11 It is also important to note that, consistent with two-thirds of the interviewees, Ernesto has a Swiss passport (other respondents are mainly European; three are undocumented). Switzerland is not a member of the European Union; European as non-European citizens must acquire an administrative permit to settle, study and work in the country. Having Swiss citizenship means, more importantly, that youths are not faced with the risk of deportation if they are caught by the police. This is not only the case for undocumented youths: since 2016, the settlement permit of “foreign criminals” (as they called by the government) can be revoked if they committed a felony (see art. 62 of the Federal Act on Foreign Nationals and Integration). In other words, citizenship has important implications regarding the youths’ vulnerability and the possibility to benefit from criminal financial gains.

12 These non-mandatory seminars are offered to young people by their social workers and are designed to help them, *inter alia*, fill in application documents, prepare for job interviews, and create working schedules.

13 Studies in Europe have shown that girls face the medical gaze (from psychiatry, in particular) much earlier and to a far greater extent than is the case for boys, whose physical and mental health problems remain seriously under-estimated and under-treated (see Amsellem-Mainguy and Dumollard 2015).

14 These minor offenses typically include violations of the Federal Act on Road and Traffic (*Loi fédérale sur la circulation routière* (“LCR”)) (e.g., driving without a license, small traffic accidents) or violations of the Federal Act on Narcotics and Psychotropic Substances (*Loi fédérale sur les stupéfiants et les substances psychotropes* (“LStup”)) (e.g., cannabis possession or use).

15 The judge is quoting the Juvenile Criminal Law Act, which sets out these three elements as fundamental in the sentencing process (art. 2 DPMin).

16 There is a sense in which some of the young people I have interviewed rationalize their delinquent career through a “strain theory” framework, and especially through the figure Merton (1938) called the “innovative” or “surviving poor.”

17 Following Messerschmidt’s (1997: 41) argument, I assert that for the young people I studied, “crime is employed to produce and sustain a specific race and class masculine identity.”
